# The Maribor consensus: report of an expert meeting on the development of performance indicators for clinical practice in ART^†^

**DOI:** 10.1093/hropen/hoab022

**Published:** 2021-07-03

**Authors:** ESHRE Clinic PI Working Group, Veljko Vlaisavljevic, Susanna Apter, Antonio Capalbo, Arianna D'Angelo, Luca Gianaroli, Georg Griesinger, Efstratios M Kolibianakis, George Lainas, Tonko Mardesic, Tatjana Motrenko, Sari Pelkonen, Daniela Romualdi, Nathalie Vermeulen, Kelly Tilleman

**Affiliations:** 1 IVF Adria Consulting D.O.O., Maribor, Slovenia; 2 Livio Fertilitetscentrum, Stockholm, Sweden; 3 Igenomix Italy, Marostica, Italy; 4 DAHFMO, Unit of Histology and Medical Embryology, Sapienza, University of Rome, Rome, Italy; 5 Wales Fertility Institute, Swansea Bay Health Board, University Hospital of Wales, Cardiff University, Cardiff, UK; 6 Societa Italiana Studi di Medicina della Riproduzione, S.I.S.Me.R. Reproductive Medicine Institute, Bologna, Emilia-Romagna, Italy; 7 Department of Gynecological Endocrinology and Reproductive Medicine, University Hospital Schleswig-Holstein, Lubeck, Germany; 8 Unit for Human Reproduction, 1st Department of ObGyn, Medical School, Aristotle University, Thessaloniki, Greece; 9 Eugonia Assisted Reproduction Unit, Athens, Greece; 10 Sanatorium Pronatal, Prague 4, Czech Republic; 11 Human Reproduction Centre Budva, Budva, Montenegro; 12 Department of Obstetrics and Gynecology, Oulu University Hospital, University of Oulu, Medical Research Center, PEDEGO Research Unit, Oulu, Finland; 13 Department of Woman and Child Health and Public Health, Woman Health Area, Fondazione Policlinico Universitario A. Gemelli IRCCS, Rome, Italy; 14 Department of Woman and Child Health, Azienda Ospedaliera Card. Panico, Tricase, Italy; 15 ESHRE Central Office, Grimbergen, Belgium; 16 Department for reproductive medicine, Universitair Ziekenhuis Gent, Gent, Belgium

**Keywords:** performance, IVF/ICSI, data collection, quality management, key performance indicators, consensus, ovarian stimulation, ART

## Abstract

**STUDY QUESTION:**

Is it possible to define a set of performance indicators (PIs) for clinical work in ART, which can create competency profiles for clinicians and for specific clinical process steps?

**SUMMARY ANSWER:**

The current paper recommends six PIs to be used for monitoring clinical work in ovarian stimulation for ART, embryo transfer, and pregnancy achievement: cycle cancellation rate (before oocyte pick-up (OPU)) (%CCR), rate of cycles with moderate/severe ovarian hyperstimulation syndrome (OHSS) (%mosOHSS), the proportion of mature (MII) oocytes at ICSI (%MII), complication rate after OPU (%CoOPU), clinical pregnancy rate (%CPR), and multiple pregnancy rate (%MPR).

**WHAT IS KNOWN ALREADY:**

PIs are objective measures for evaluating critical healthcare domains. In 2017, ART laboratory key PIs (KPIs) were defined.

**STUDY DESIGN, SIZE, DURATION:**

A list of possible indicators was defined by a working group. The value and limitations of each indicator were confirmed through assessing published data and acceptability was evaluated through an online survey among members of ESHRE, mostly clinicians, of the special interest group Reproductive Endocrinology.

**PARTICIPANTS/MATERIALS, SETTING, METHODS:**

The online survey was open for 5 weeks and 222 replies were received. Statements (indicators, indicator definitions, or general statements) were considered accepted when ≥70% of the responders agreed (agreed or strongly agreed). There was only one round to seek levels of agreement between the stakeholders.

Indicators that were accepted by the survey responders were included in the final list of indicators. Statements reaching less than 70% were not included in the final list but were discussed in the paper.

**MAIN RESULTS AND THE ROLE OF CHANCE:**

Cycle cancellation rate (before OPU) and the rate of cycles with moderate/severe OHSS, calculated on the number of started cycles, were defined as relevant PIs for monitoring ovarian stimulation. For monitoring ovarian response, trigger and OPU, the proportion of MII oocytes at ICSI and complication rate after OPU were listed as PIs: the latter PI was defined as the number of complications (any) that require an (additional) medical intervention or hospital admission (apart from OHSS) over the number of OPUs performed. Finally, clinical pregnancy rate and multiple pregnancy rate were considered relevant PIs for embryo transfer and pregnancy. The defined PIs should be calculated every 6 months or per 100 cycles, whichever comes first. Clinical pregnancy rate and multiple pregnancy rate should be monitored more frequently (every 3 months or per 50 cycles). Live birth rate (LBR) is a generally accepted and an important parameter for measuring ART success. However, LBR is affected by many factors, even apart from ART, and it cannot be adequately used to monitor clinical practice. In addition to monitoring performance in general, PIs are essential for managing the performance of staff over time, and more specifically the gap between expected performance and actual performance measured. Individual clinics should determine which indicators are key to the success in their organisation based on their patient population, protocols, and procedures, and as such, which are their KPIs.

**LIMITATIONS, REASONS FOR CAUTION:**

The consensus values are based on data found in the literature and suggestions of experts. When calculated and compared to the competence/benchmark limits, prudent interpretation is necessary taking into account the specific clinical practice of each individual centre.

**WIDER IMPLICATIONS OF THE FINDINGS:**

The defined PIs complement the earlier defined indicators for the ART laboratory. Together, both sets of indicators aim to enhance the overall quality of the ART practice and are an essential part of the total quality management. PIs are important for education and can be applied during clinical subspecialty.

**STUDY FUNDING/COMPETING INTEREST(S):**

This paper was developed and funded by ESHRE, covering expenses associated with meetings, literature searches, and dissemination. The writing group members did not receive payment.

Dr G.G. reports personal fees from Merck, MSD, Ferring, Theramex, Finox, Gedeon-Richter, Abbott, Biosilu, ReprodWissen, Obseva, PregLem, and Guerbet, outside the submitted work. Dr A.D. reports personal fees from Cook, outside the submitted work; Dr S.A. reports starting a new employment in May 2020 at Vitrolife. Previously, she has been part of the Nordic Embryology Academic Team, with meetings were sponsored by Gedeon Richter. The other authors have no conflicts of interest to declare.

**DISCLAIMER:**

This document represents the views of ESHRE, which are the result of consensus between the relevant ESHRE stakeholders and where relevant based on the scientific evidence available at the time of preparation.

The recommendations should be used for informational and educational purposes. They should not be interpreted as setting a standard of care, or be deemed inclusive of all proper methods of care nor exclusive of other methods of care reasonably directed to obtaining the same results. They do not replace the need for application of clinical judgment to each individual presentation, nor variations based on locality and facility type.

Furthermore, ESHREs recommendations do not constitute or imply the endorsement, recommendation, or favouring of any of the included technologies by ESHRE.

WHAT DOES THIS MEAN FOR PATIENTS?

Performance indicators (PIs) are seen as a valid way to check that the healthcare provided is high in quality and operates within acceptable limits. Although there already is a list of PIs for the laboratories working on infertility and ART (such as IVF, ICSI), a list of PIs for the ART clinical work is lacking. This paper looks at PIs for ART clinical work for different steps of the ART process, starting from fertility workup and diagnosis, extending to ovarian stimulation and oocyte pick-up (OPU), and ending with embryo transfer and the birth of a healthy baby. After consensus was reached in the working group, the PIs were sent through an online survey to find agreement within a group of medical experts. Finally, six PIs were defined: cycle cancellation rate (before OPU), rate of cycles with moderate/severe ovarian hyperstimulation syndrome, the proportion of mature oocytes at ICSI, complication rate after OPU, clinical pregnancy rate, and multiple pregnancy rate.

The working group concluded that it was important for each clinic and/or individual doctor to monitor and check their own performance regularly using these PIs. The PIs are an important step towards improving the outcomes of ART and achieving high-quality ART services.

## Introduction

Treating infertility with ART is a complex process combining clinical work and laboratory procedures. The process involves different steps, including hormonal stimulation and monitoring of the associated ovarian response, oocyte pick-up (OPU), fertilization, embryo development, and/or cryopreservation in the laboratory, and intrauterine embryo transfer (ET), leading to implantation, pregnancy and eventually, the birth of a healthy child (Australian In-Vitro Fertilization Collaborative Group, 1988; Dyer *et al.*, 2016; Wilkinson *et al.*, 2016).

The complexity of the process is reflected in the large number of data that can be generated from an ART cycle, as shown and collected in national and international data registries (The European IVF-monitoring Consortium for the European Society of Human Reproduction and Embryology *et al.*, 2020). Currently, there are no unified indicators to measure success in the clinical part of an ART cycle of which the main aim is to ensure the safety of the procedure for the patient and the new-born, as well as its effectiveness towards the key objective, i.e. the birth of a healthy singleton child. Recently, performance indicators (PIs) have been defined and agreed upon for the laboratory part of the ART cycle (ESHRE Special Interest Group of Embryology and Alpha Scientists in Reproductive Medicine, 2017).

PIs are objective measures for evaluating critical healthcare domains (patient safety, effectiveness, equity, patient-centeredness, timeliness, and efficiency) (Institute of Medicine Committee on Quality of Health Care in America, 2000). Systematic monitoring of PIs is considered part of the total quality management system (De los Santos *et al.*, 2016) and is gaining interest in clinical practice. PIs can be monitored for internal auditing (quality control and assurance) and for contributing to a continuous process of clinical performance improvement. Additionally, PIs can be used for external reporting with possible consequences for health care service funding or reimbursement systems. PIs can also focus on patient-centeredness and are, among others, the competence of the staff in being empathic, information provision before, during, and after treatment, and waiting times (Dancet *et al.*, 2010, 2013). Although these are interesting indicators to monitor, they fall outside the scope of the current paper.

Focussing only on the predefined objective of assisted reproduction, i.e. the live birth of a healthy child, as a proxy for quality may be problematic as reaching this objective is known to be affected by factors other than the quality of care (Lilford *et al.*, 2007). Specifically for ART, these factors include, but are not limited to, biological and clinical factors impacting on ovarian response and embryo development, or resulting in implantation failure and pregnancy complications. Furthermore, ART process outcomes are not only impacted by the inherent limitation of natural human conception, but also they may be influenced by clinical treatments and/or laboratory processes (Pirtea *et al.*, 2020). Measuring performances in different steps of the ART process, and acting on them accordingly, will reduce variability and aid in controlling the latter processes better, which results in improving outcomes in assisted reproduction.

Monitoring laboratory performance is crucial in any clinic performing ART (De los Santos *et al.*, 2016). PIs for the laboratory have been previously addressed (Lilford *et al.*, 2007; ESHRE Special Interest Group of Embryology and Alpha Scientists in Reproductive Medicine, 2017; Franco *et al.*, 2017; Fabozzi *et al.*, 2020). Performance is checked regularly on both operator and procedural levels (ESHRE Special Interest Group of Embryology and Alpha Scientists in Reproductive Medicine, 2017), as this is the only way to demonstrate, analyse, and improve outcomes. An important indicator for measuring the overall efficiency of a culture system as a whole is the implantation rate (IR) (ESHRE Special Interest Group of Embryology and Alpha Scientists in Reproductive Medicine, 2017). There has been debate on the laboratory PIs stated in the Vienna consensus, especially on the threshold levels of the PIs to define the competence of laboratories (Lopez-Regalado *et al.*, 2018). Indeed, defining universally accepted threshold indicator values is difficult. It is nevertheless important to use PIs to monitor performance of your staff and your centre based on your patient population, within your specific region where practice can be restricted by legislation. The aim of consensus papers on PIs is to speak a ‘common language’ and analyze the same indicators using the same formulas. It is the very basis to start discussing and improving laboratory practices, and tracking laboratory PIs has been shown to be very relevant when planned or unexpected changes in procedures occur (Hammond and Morbeck, 2019): the latter study eloquently shows the relation between laboratory PIs and more clinically related outcomes such as IR. IR, like other indicators, is indeed used to measure laboratory performance, but is influenced by a range of factors that are not always controlled for by the laboratory. Treatment outcome is highly influenced by both patient-related parameters (e.g. age, lifestyle factors, indication for fertility treatment, BMI, the quality of the gametes, uterine receptivity) and clinical practice (e.g. stimulation protocols, transfer policies, the competence of the clinical staff). The patient characteristics set the conditions for the treatment while the association between the clinical and laboratory work creates the conditions for the results. Unsurprisingly, this association is strong and two-way; indicating that ART is a team effort at its best. In this Vienna consensus, two reference indicators (proportion of oocytes recovered and proportion of mature [metaphase II (MII)] oocytes at ICSI) were defined that are closely related to the quality of the clinical practice (ESHRE Special Interest Group of Embryology and Alpha Scientists in Reproductive Medicine, 2017).

Information on clinical PIs is even more scarce and there is definitely no broad consensus among ART clinicians on which PIs should be measured, let alone the indicator threshold values. The current paper, therefore, aims: first to determine a set of PIs for clinical work in ART; and second to determine clear definitions of these PIs with limits of acceptable competence levels to be used in the quality management system of each ART centre. The current PIs for clinical work in ART are complementary to the ART laboratory PIs published in 2017 (ESHRE Special Interest Group of Embryology and Alpha Scientists in Reproductive Medicine, 2017) and together they aim to improve the overall quality and the outcome of ART practice.

## Materials and methods

The current paper was developed based on the methodology for the development of ESHRE good practice recommendations (Vermeulen *et al.*, 2019). All co-ordinators of the ESHRE special interest groups (SIGs) and relevant ESHRE committees and other experts representing different regions within Europe were invited to join the working group.

**Figure 1.**
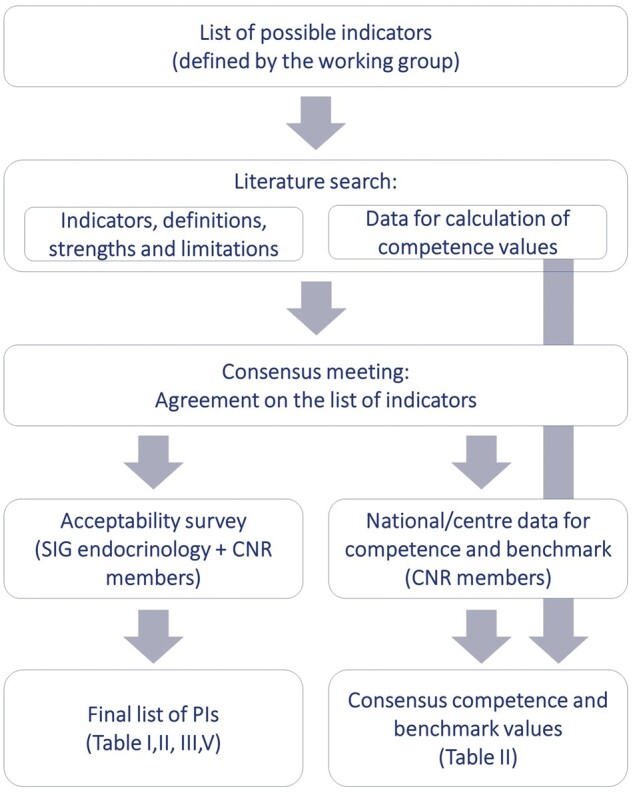
**Methodology for the paper on the development of performance indicators for clinical practice in ART.** CNR, committee of national representatives; PI, performance indicator; SIG, special interest group.

The working group composed a list of possible indicators and drafted their definitions (Fig. 1). A meeting was organized to reach a consensus on the indicators, after which evidence was collected from Pubmed on the strengths and limitations of each proposed indicator. The working group was split into six subgroups, each investigating the relevance of the papers and summarizing the evidence on a specific set of indicators. For selected indicators, poor, normal, and high responders to ovarian stimulation were discussed separately. Poor response was defined according to the Bologna criteria as ≤ 3 follicles on day of oocyte maturation trigger and/or ≤ 3 oocytes obtained characterize a low response (Ferraretti *et al.*, 2011; Zegers-Hochschild *et al.*, 2017). High response was defined as more than 18 follicles ≥11 mm in size on day of oocyte maturation trigger and/or 18 oocytes collected (The ESHRE Guideline Group on Ovarian Stimulation *et al.*, 2020). The values of these indicators from published data were calculated through weighted average analysis and only studies defining ovarian response as such were taken into consideration.

The (narrative) summary of evidence and final list of PIs were discussed and agreed upon during a 2-day meeting in Maribor (November 2019). The results of the meeting (i.e. summary statements, PIs per section, and general recommendations) were used as the basis for a survey with the aim of assessing acceptability on a 5-point Likert Scale. The online survey (developed in SurveyMonkey) was distributed on 07/05/2020 to the members of the SIG Reproductive Endocrinology (2168 e-mail addresses, >80% clinicians) and the members of the ESHRE committee of national representatives (CNR) (n = 68). The survey was open for 5 weeks. Statements (indicators, indicator definitions, or general statements) were considered accepted when 70% of the responders agreed (agreed or strongly agreed). Only indicators that were accepted by the survey responders were included in this consensus paper (Tables I and II). Other statements were considered ‘debatable’ and were further discussed in the manuscript but not listed in the table of PIs.

**Table I:** Performance indicators for clinical practice in ART.

Performance indicator	Calculation
**Cycle cancellation rate (before OPU) (%CCR)**	Nr of cycles cancelled before OPU × 100
	
	Nr of started cycles

**Rate of cycles with moderate/severe OHSS (% mosOHSS)**	Nr of cycles with moderate to severe OHSS × 100
	
	Nr of started cycles

**Proportion of MII oocytes at ICSI (%MII)**	Nr of MII oocytes at ICSI × 100
	
	Nr of cumulus-oocyte complexes retrieved

**Complication rate after OPU (%CoOPU)**	Nr of complications (any) that require an (additional) medical intervention or hospital admission (apart from OHSS) × 100
	
	Nr of OPUs performed

**Clinical pregnancy rate (%CPR)**	Nr of pregnancies (diagnosed by ultrasonographic visualization of one or more gestational sacs or definitive clinical signs of pregnancy) × 100
	
	Nr of embryo transfer cycles

**Multiple pregnancy rate (%MPR)**	Nr of pregnancies with more than one embryo or foetus × 100
	
	Nr of pregnancies

A started cycle is considered an ART cycle in which ovarian stimulation was initiated.

OPU, oocyte pick up; OHSS, ovarian hyperstimulation syndrome; MII, mature oocyte.

**Table II:** Overview of competence and benchmark values for the performance indicators.

Performance indicator		Competence value^1^ (%)					Benchmark value^1^ (%)		
		Calculated from published data		Calculated from data reported by CNR members		Consensus value	Calculated from data reported by CNR members		Consensus value
	POPULATION	Mean	95% CI	Mean	95% CI		Mean	95% CI	
**Cycle cancellation rate (before OPU) (%CCR)**	Reference population	5	4–6	6.29	4.55–8.03	6	3.75	2.88–4.62	3.5
	
	Poor responders	40	30–49	28.86	14.51–43.21	40	20.00	10.04–29.96	20
	
	Normal responders	20	11–29	11.83	3.56–20.11	20	6.93	2.55–11.30	7
	
	High responders	3	1–4	2.50	1.93–3.07	3	1.50	−0.03 to 3.03	1.5

**Rate of cycles with moderate/severe OHSS (with antagonist protocol) (%mosOHSS)**	Reference population			1.52	0.42–2.62	1.5	0.61	0.17–1.06	0.5
	
	Normal responders	3	1–5	1.44	0.44–2.44	3	0.44	0.04–0.85	0.5
	
	High responders	2	0–5	2.89	1.03–4.74	3	1.64	−0.06 to 3.35	1.5

**Rate of cycles with moderate/severe OHSS (with agonist protocol) (%mosOHSS)**	Reference population			2.59	−0.51 to 5.68	2.5	1.13	0.12–2.13	1
	
	Normal responders	6	3–11	3.70	0.87–6.52	6	2.08	0.31–3.85	2
	
	High responders	11	4–20	7.63	3.66–11.61	11	5.83	2.23–9.43	5.5

**Proportion of MII oocytes at ICSI (%MII)**	Reference population			74.13	67.51–81.24	74	81.25	72.67–89.83	75–90^2^

**Complication rate after OPU (%CoOPU)**	Reference population	0.2		0.36	0.10–0.62	0.5	0.19	−0.09 to 0.46	0.1

**Clinical pregnancy rate (%CPR)**	Reference population			32.24	29.27–35.21	na^3^	35.50	26.32–51.35	na^3^

**Multiple pregnancy rate (%MPR)**	Reference population			12.82	8.36–17.28	13	7.71	2.69–12.74	7.5

Values were deduced from published data and calculated (mean and 95% CI) from data collected through the members of the committee of national representatives (CNR).

^1^ The competence value is the minimum expected value (i.e. the value that any clinic should be able to achieve). The benchmark value is the aspirational value (i.e. the value that can be employed as a best practice goal).

^2^ Consistent with the Vienna consensus (ESHRE Special Interest Group of Embryology and Alpha Scientists in Reproductive Medicine, 2017).

^3^ Owing to heterogeneity, data inconsistency, absence of data validation and errors in data collection, it was deemed impossible to define competence and benchmark values for embryo transfer (ET) outcomes that could comprehensively apply to all European clinics. Competence and benchmark values should be set for a specific local context, for instance from the data reported to the European IVF-monitoring Consortium for the same country.

Additionally, the CNR members were asked to provide minimum expected, or competence, values (i.e. values that any clinic should be able to achieve), and aspirational, or benchmark, values (i.e. values that can be employed as a best practice goal) for the different PIs based on national data (if available), or data from their own centre. Mean and 95% CI were calculated from the collected competence values. If a range, rather than a fixed number was suggested, the upper or lower value was used, depending on the indicator. The upper value was used for all indicators aiming at maximum values, i.e. proportion of MII oocytes at ICSI (%MII) and clinical pregnancy rate (%CPR). The calculated values from the literature search and those reported by the CNR members were used as a basis for the working group to define appropriate competence and benchmark values for each indicator. The final draft of the paper was discussed among the working group members before publication.

## Results

### Recommendations of the expert panel

This paper describes PIs in four steps of a standard ART process: diagnosis of infertility and indications for ART treatment; ovarian stimulation; monitoring of ovarian stimulation, trigger, and OPU; and ET and pregnancy. In addition, indicators for training and competence are described (Supplementary Fig. S1).

The survey, which consisted of a total of 31 statements and formulas on PIs described for each step of the ART process, received 222 replies. Overall, an agreement was reached for 83.87% of the statements, meaning at least 70% of the survey responders (strongly) agreed with the statement (Supplementary Table SI). Agreement on statements of diagnosis and indications, ovarian stimulation, monitoring/trigger/OPU, ET/pregnancy were found in 80.00%, 85.71%, 85.71%, and 87.50%, respectively. The PIs achieving 70% agreement and the formulas for calculating them are summarized in Table I.

Suggestions for competence (minimum expected) and benchmark (aspirational) values for the different PIs were received from 11 countries (Bulgaria, Denmark, Germany, Hungary, Lithuania, Montenegro, Poland, Portugal, Russia, Slovenia, UK). The competence and benchmark values based on the data provided by the national representatives combined with the competence values calculated from published data are available in Table II.

In the Vienna consensus on laboratory PIs, a reference population was defined as female patients <40 years old, using own fresh oocytes, ejaculated spermatozoa (fresh or frozen), any insemination method (i.e. routine IVF and ICSI), and no preimplantation genetic testing (PGT). Unless specified differently, this paper uses the same reference population. For PIs related to ovarian stimulation and ovarian response monitoring (cycle cancellation rate and rate of cycles with moderate/severe OHSS), a calculation in populations stratified according to ovarian response (poor, normal, and high response) is considered relevant (Ferraretti *et al.*, 2011, The ESHRE Guideline Group on Ovarian Stimulation *et al.*, 2020). For PIs related to ET and pregnancy (%CPR and %MPR), different possible subgroups have also been defined (see below).

Regarding the frequency of data collection and analyzing trends in the PIs for ovarian stimulation, monitoring, trigger and OPU, it was concluded that PIs should be calculated at least every 6 months, or per 100 cycles, whichever comes first. For the PIs for ET, it was concluded that they should be calculated every 3 months, or per 50 cycles, whichever comes first. Reporting on a lower number of cycles is possible, but this will result in larger CIs and impact the analysis and interpretation of results (Table III).

**Table III:** Frequency of reporting.

Performance indicator	Suggested frequency of analysis/reporting
**Cycle cancellation rate (before OPU) (%CCR)**	
	
**Rate of cycles with moderate/severe OHSS (%mosOHSS)**	Calculate every 6 months, or per 100 cycles, whichever comes first.
	
**Proportion of MII oocytes at ICSI (%MII)**	
	
**Complication rate after OPU (%CoOPU)**	

**Clinical pregnancy rate (%CPR)**	Calculate every 3 months, or per 50 cycles, whichever comes first.
	
**Multiple pregnancy rate (%MPR)**	

### Monitoring performance in ART clinical practice

In ART, standardization of treatment is difficult and there is a huge difference in control of clinical decision-making and competence, partly owing to differences in training programmes and organization of education among countries, and also owing to differences in legislation. Centres should have written policies and protocols for access and treatment, and for all procedures performed.

The PIs defined in the current paper are relevant parameters to assess performance in ART clinical practice and should be routinely monitored and reported according to a defined frequency (Table III). Individual clinics should decide whether it is relevant and practical to subdivide their results into specific patient groups for PI determination and which indicators are key to the success in their organization. As such, individual clinics will be able to define their own set of key PIs (KPIs). The strategic organizational management, the clinical practice, the treatments performed and the scale of the organization all impact on the indicator and their values. The idea of PIs is to use and monitor them within your practice and to act upon fluctuations. PIs are there to improve your own practice and to set reachable goals for your practice. Too often PIs are only used to remediate and find root causes upon dropping PI values. However, with a periodic rise in PIs, it is equally important to investigate the reasons for the improvement and more importantly, to try to keep the improved process and outcomes for a longer period.

### PIs for diagnosis of infertility and indications for ART treatment

Infertility is, in many instances, not a definite diagnosis but rather a biological phenomenon on a continuous scale from normal fertility to subfertility to infertility and, rarely, irreversible sterility. Furthermore, infertility can involve several individuals, which adds to the complexity and the multitude of biological factors to be considered when establishing a diagnosis and suggesting an ART treatment (IVF and/or ICSI).

As a first step, a thorough medical history is collected and a physical examination is performed with the aim of finding definite or potential causal factors for infertility and in turn tailoring management accordingly (Practice Committee of the American Society for Reproductive Medicine, 2012).

In the female patient, the physical examination should include a 2-dimensional transvaginal ultrasound (US). Further assessment can be performed depending on indication and includes 3-dimensional US, hystero-contrast-sonography (HyCoSy) or hysterosalpingography (for assessment of the fallopian tubes), and/or hysteroscopy or laparoscopy in distinct cases where co-morbidities might be present. Ovulation and its disorders should be examined by assessing the menstrual calendar and by laboratory testing. Ovarian reserve assessment may be considered useful in addition to female age as a predictor of ovarian response and treatment outcome (The ESHRE Guideline Group on Ovarian Stimulation *et al.*, 2020).

For the male patient, a semen analysis based on World Health Organization standards should be performed (World Health Organization, 2010). Based on this analysis, a full evaluation by an andrologist or other specialist in male reproduction may be required. Further evaluation of the male patient should also be considered in the case of a heterosexual couple with unexplained infertility or when there is a treated female factor and persistent infertility (Barratt *et al.*, 2017).

Established indications for ART are: tubal damage or blockage; severe male factor infertility; unexplained infertility (selected cases); severe endometriosis (Dunselman *et al.*, 2014); genetic disorders indicating PGT; medical conditions requiring oocyte or embryo donation, or use of a gestational carrier (surrogacy); medical indications for fertility preservation (male and female) (ESHRE Guideline Group on Female Fertility Preservation *et al.*, 2020); infertility or subfertility related to pathologies with an immunologic origin; or infertility or subfertility attributed to low ovarian reserve or advanced female age.

Once the indication for ART is confirmed, the prognosis but also the risk profile related to the treatment and the subsequent pregnancy should be assessed, and patients must be counselled accordingly. The following, non-exhaustive, list illustrates some typical aspects to be considered:

- *Female age*: increasing age is correlated with reduced ART success and increased obstetrical risk in terms of early pregnancy loss, preterm delivery, gestational diabetes, hypertensive disorders, intrauterine growth retardation (IUGR), peripartum haemorrhage, and caesarean section (Lean *et al.*, 2017).
- *BMI*: a bodyweight under or above the normal range (considered 18.5–24.9 kg/m^2^) is correlated with increased procedural risks (such as adverse events during OPU), obstetric complications, and perinatal risks (e.g. IUGR, macrosomia, gestational diabetes, malformations, and others) (Liu *et al.*, 2016; ESHRE Working Group on Ultrasound in ART *et al.*, 2019).
- *Concomitant disorders*: there may be an interaction of ART treatment and/or pregnancy with concomitant disorders, such as endocrine-metabolic disorders (e.g. diabetes, thyroid disorders), gynaecological conditions (e.g. endometriosis), auto-immune disorders (e.g. multiple sclerosis, systemic lupus erythematosus), thrombophilia, previous abdominal surgery, single organ or systemic diseases. In women with concomitant disorders, the risks of the ART treatment, the fitness for pregnancy, and required obstetric monitoring should be assessed, where needed in a multidisciplinary context.
- *Transmission risk for infectious diseases.*

In addition to medical indications, ART treatments are being performed in many countries and centres (Adamson *et al.*, 2018) based on empirical grounds, patient request, and market forces as well as by the extension of ART techniques to fertility preservation, PGT, and even other indications, such as oocyte cryopreservation for age-related fertility loss (Evers, 2016; Adamson *et al.*, 2018). Infertile individuals may opt for ART treatment in the absence of well-established medical indications (Evers, 2016), even though it has been shown that tailored expectant management in good prognosis patients does not negatively affect the chance of pregnancy, while, conversely, it reduces overtreatment and the associated risks, burden, and costs (Kersten *et al.*, 2015). Furthermore, ICSI treatment has grown disproportionally over IVF treatment (De Geyter *et al.*, 2018; The European IVF-monitoring Consortium for the European Society of Human Reproduction and Embryology *et al.*, 2020).

The decision on whether to initiate or proceed with ART treatment must first and foremost be a medical one, based on the results of the diagnostic workup and assessment of risks, and in line with patient preferences, national legislation, reimbursement schemes, availability of services and/or socio-cultural aspects. In the absence of PIs for this section, the following recommendations were formulated and achieved agreement (Supplementary Table SI):

- A diagnostic fertility workup should be performed to assess the chance of natural conception, to identify causal factors for infertility, to predict the chance of success of ART treatments, and to detect risk factors for complications during ART treatment or pregnancy.
- In decision-making with regards to ART, four treatment dimensions should be considered: burden, effectiveness, safety, and costs (Dancet *et al.*, 2014). Expected benefits should be weighed against not only the risks and burden of treatment, including complications, but also the health of the subsequent pregnancy and the child.

*In the survey, the stakeholders accepted the above-mentioned recommendations. A third statement reading: ‘ART should only be considered for cases with no alternative treatment of less invasiveness, burden, risks and costs; was borderline debatable, but finally not accepted: 69.41% of respondents agreed and 14.16% disagreed (16.44% neither agreed nor disagreed). Although the statement represents a general principle in medicine, patient-centeredness and preference are highly relevant in ART decision-making.*

### Indicators for ovarian stimulation

#### Cycle cancellation rate prior to OPU

Cancellation of an ART cycle is an unexpected outcome that can occur prior to or after OPU. Cycle cancellation after OPU may result from zero oocytes being identified after oocyte retrieval (see also the discussion of the rate of no oocytes retrieved or occurrence of empty follicle syndrome), failed fertilization, or poor embryo development. Based on published data from 14 studies, the cancellation rate between OPU and ET is estimated to be 14% (weighted average). Cycle cancellation between OPU and ET is not considered a good indicator for clinical work since it can be related to biological factors (such as failure of sperm to fertilize), factors related to the capacity of the oocyte to be activated and fertilized, or factors associated with the clinic’s ET strategy (such as blastocyst transfer regardless of the number of oocytes or embryos available). Cancellation between OPU and ET related to laboratory performance can be monitored by other PIs (e.g. failed fertilization rate, embryo development rate). Therefore, the clinical PI cycle cancellation should focus on cancellation before OPU only.

Cycle cancellation before OPU can be attributed to poor response to ovarian stimulation, premature ovulation, or errors in taking medications, but it may also be influenced by local reimbursement policy or patient preferences. The cycle cancellation rate for 2016 was calculated from the reported number of cycles and the number of aspirations in the European IVF-monitoring Consortium (EIM) data (The European IVF-monitoring Consortium for the European Society of Human Reproduction and Embryology *et al.*, 2020). Overall, the EIM reported 42 626 cancellations in 538 788 cycles (based on countries for which data were available), resulting in a cancellation rate of 7.91% (95% CI 7.84–7.98). Cycle cancellation rate before OPU is dependent on the population treated, with estimations ranging from 3% in high responders and 20% in a general population to 40% in poor responders. The EIM report does not provide data for different types of populations treated, and the reported cancellation rates are likely an underestimation of the true ones (The European IVF-monitoring Consortium for the European Society of Human Reproduction and Embryology *et al.*, 2020).

*In the survey, cycle cancellation before OPU was agreed to be a relevant parameter to assess performance in ovarian stimulation. Its suggested definition of the number of cycles cancelled prior to OPU over the number of started cycles was accepted, as was the suggestion to calculate the PI separately for poor, normal, and high responders.*

#### OHSS rate

Excessive ovarian response is characterized by the growth of a large number of follicles, leading to an increased probability of developing OHSS. Several follicle thresholds have been proposed as critical for predicting the occurrence of OHSS, for instance, 14 follicles larger than 11 mm for the general population (Papanikolaou *et al.*, 2005), or >20 follicles larger than 11 mm for patients without polycystic ovary syndrome (PCOS) or non-poor responder patients (Griesinger *et al.*, 2016).

For many years the only available GnRH analogues for suppressing the LH surge were GnRH agonists. The use of GnRH agonists in combination with gonadotrophins in ovarian stimulation was associated with the development of OHSS in a significant proportion of patients. OHSS was considered a price to be paid for obtaining multiple oocytes and creating multiple embryos allowing the selection of the best ones for transfer (Abramov *et al.*, 1999). The incidence of OHSS was reduced significantly with the availability of GnRH antagonists (Al-Inany *et al.*, 2011) and was almost eliminated with the replacement of hCG with GnRH agonist and freezing all embryos in high-risk patients (Tarlatzis and Bosdou 2017; Vlaisavljevic *et al.*, 2017). Nowadays, the goal is to completely avoid this iatrogenic in nature complication, and severe OHSS, the most dramatic of its complications, must never occur (Devroey *et al.*, 2011). In practice, however, it is not always feasible to apply an antagonist protocol with GnRH agonist triggering and freezing of all embryos in high-risk patients, as well as to adequately identify these patients and treat them accordingly (Broekmans, 2019). The probability of OHSS depends on the type and intensity of the stimulation applied but also on the type of population treated (Fernandez-Sanchez *et al.*, 2019). Thus, an estimation of the incidence of OHSS expected under various stimulation protocols and types of populations is necessary to evaluate a clinician’s performance in this respect.

Published data from registries and meta-analyses were used to estimate the occurrence of severe OHSS, with significant limitations. The OHSS incidence reported by the EIM is based on OHSS Grades 3, 4, and 5, and OHSS requiring hospitalization (The European IVF-monitoring Consortium for the European Society of Human Reproduction and Embryology *et al.*, 2020). The pooled estimated incidence of OHSS reported for each country is 0.210% (95% CI 0.201–0.220), which is considered an underestimation of its real incidence. The meta-analysis by Lambalk et al. (2017) examined OHSS per woman randomized without discriminating between different degrees of OHSS severity and, thus, it was considered as not clinically useful. The data provided by the Cochrane meta-analysis refer to moderate/severe OHSS in GnRH agonist and antagonist cycles (Al-Inany *et al.*, 2011, 2016). The estimated incidence of moderate/severe OHSS was 2.14% (95% CI 1.156–5.36) for infertile women undergoing ovarian stimulation with a GnRH antagonist protocol and 6.43% (95% CI 2.75–11.33) for women with a GnRH agonists protocol. For women with PCOS, the incidences of moderate/severe OHSS were 2.94% (95% CI 0.41–4.77) and 10.61% (95% CI 3.82–19.95) for the GnRH antagonist and GnRH agonist protocol, respectively. The limitations regarding these data are that: the definition of OHSS varied across studies included in the meta-analysis (Al-Inany *et al.*, 2016); the reported data refer to moderate/severe OHSS rather than severe OHSS only, which is considered to be more clinically relevant; and no distinction could be made between early and late OHSS (Al-Inany *et al.*, 2011, 2016).

By combining the results from available trials evaluating freeze-all strategies or donor patients (13 studies, 1908 patients) in which GnRH agonist was used for triggering final oocyte maturation and no luteal phase support was administered (freeze-all strategy), the estimated incidence of severe OHSS was 0.00 (95% CI 0.00–0.00) (Tarlatzis and Bosdou, 2017), although sporadic cases of OHSS under this approach have also been reported (Kol, 2011).

In summary, if GnRH agonists are used for ovarian stimulation, the expected incidence of moderate/severe OHSS is 6.43% and 10.61% in the regular population and in patients with PCOS, respectively. When GnRH antagonists are used, the expected incidences of moderate/severe OHSS are 2.94% and 2.14% in the regular population and in patients with PCOS, respectively. A further reduction of OHSS incidence, by replacing hCG with GnRH agonist and not performing a fresh transfer, has been reported (Tarlatzis and Bosdou, 2017).

*In the survey, the indicator rate of cycles with moderate/severe OHSS was considered a relevant parameter to assess performance in ovarian stimulation, and its suggested definition of the number of cycles with moderate to severe OHSS over the number of started cycles was acceptable. Competence and benchmark values were defined for all patients, normal and high responders, and for agonist and antagonist protocols. In the survey, respondents commented on the lack of relevance of calculating the parameter for poor responders, which was corrected.*

### Indicators for monitoring of ovarian response, trigger, and OPU

#### Rate of no oocytes retrieved or occurrence of empty follicle syndrome

Empty follicle syndrome (EFS) is defined as the complete failure to retrieve oocytes during OPU, despite apparently normal development of ovarian follicles and appropriate oestradiol production by granulosa cells (Coulam *et al.*, 1986). Two variants of EFS have been described: the ‘genuine’ form, which occurs after a correct ovulation trigger (by hCG or GnRH-analogue), and the ‘false’ form, which is associated with low hCG or LH levels and can be attributed to a (human) error in the administration of the trigger or, for example, a result of rapid metabolic clearance in the patient. However, threshold levels for hCG or circulating LH and progesterone, required to discriminate between ‘genuine’ and ‘false’ EFS, are not standardized. Besides, it is stipulated that EFS does not represent a permanent pathophysiological condition because many cases occur sporadically. The existence of EFS has been questioned, claiming that when ovarian stimulation is adequately carried out and the ovulation trigger is correctly administered, the total failure to retrieve oocytes represents a sporadic event rather than a true syndrome (van Heusden *et al.*, 2008). Advice on troubleshooting during OPU, and specifically on what to do when no oocytes are retrieved, has been previously covered in an ESHRE good practice paper (ESHRE Working Group on Ultrasound in ART *et al.*, 2019).

#### Oocyte retrieval rate

Oocyte retrieval rate (ORR) or the proportion of oocytes recovered (in stimulated cycles) is defined as the number of oocytes retrieved during OPU over the number of follicles on the day of trigger (ESHRE Special Interest Group of Embryology and Alpha Scientists in Reproductive Medicine, 2017). ORR is a measure of the quality of patient monitoring and may be influenced by the timing of the trigger while it is a useful reference indicator for the laboratory (Van Voorhis *et al.*, 2010; ESHRE Special Interest Group of Embryology and Alpha Scientists in Reproductive Medicine, 2017).

Traditionally, the timing of hCG administration is based on the size of the leading follicle(s) (Bosdou *et al.*, 2015). The association of cumulative live birth rate (LBR) with the number of oocytes retrieved is well established (Sunkara *et al.*, 2011; Drakopoulos *et al.*, 2019). It is suggested that larger follicles may be more likely to yield mature oocytes, although it has been demonstrated that even small follicles may yield oocytes capable of fertilization, cleavage, implantation and pregnancy (Jones *et al.*, 1984; ESHRE Working Group on Ultrasound in ART *et al.*, 2019).

Concerning the number of follicles, these can be measured and counted on the day of triggering final oocyte maturation. The relevance of follicle size as a triggering criterion has not been sufficiently studied (The ESHRE Guideline Group on Ovarian Stimulation *et al.*, 2020). In addition, the decision on the timing of triggering in relation to follicle size is multi-factorial, taking into account the size of the growing follicle cohort, the hormonal data on the day of trigger, duration of stimulation, patient burden, financial costs, experience of previous cycles and organizational factors for the centre. Clinicians may choose the follicle size upon which final oocyte maturation is triggered on a case-by-case basis. Most often, final oocyte maturation is triggered when several of the leading follicles are between 16 and 22 mm. Follicle size is always an approximation, as it is not practical to perform measurements of every single punctured follicle during OPU as this would increase the time of the procedure and might increase the risks associated with it (e.g. injury, haemorrhage, infection) (Van Voorhis *et al.*, 2010; ESHRE Special Interest Group of Embryology and Alpha Scientists in Reproductive Medicine, 2017).

ORR has been defined as the number of oocytes retrieved over the number of follicles on the day of trigger (ESHRE Special Interest Group of Embryology and Alpha Scientists in Reproductive Medicine, 2017). In the Vienna consensus, no values on follicle size were included in the denominator, and suggestions have been made with a high variation ranging from 10 mm to 15 mm. It is clear that changing the denominator, and more specifically the cut-off follicle size, will largely affect the values for the indicator. A study from Bosdou et al. (2015) calculated ORR as the number of oocytes over the number of follicles ≥11 mm and reported a mean ORR of 62.5% (95% CI 56.1–70.2). The authors also reported an association with maternal age, i.e. decreasing ORR with increasing age (Bosdou *et al.*, 2015). In clinical practice, there is substantial variety; clinicians may puncture different sizes of follicles. It was recently suggested that small follicles (<10 mm) can be left un-punctured (to avoid the collection of immature eggs) unless there is a high risk of OHSS (ESHRE Working Group on Ultrasound in ART *et al.*, 2019). A decision to puncture smaller follicles is considered a clinical decision, which should consider patient safety.

*Although from the survey ORR is considered a relevant PI to be calculated, the suggested definition, i.e. the number of oocytes retrieved over the number of follicles (>10 mm) on the day of trigger, was judged as debatable. Comments were made on limitations related to the technical feasibility and subjectivity of counting follicles, especially as there is no clear agreement or exact measurement of the follicle size. ORR was not included as a consensus PI.*

#### Proportion of MII oocytes at ICSI

The proportion of MII oocytes at ICSI or rate of mature oocytes was categorized as a reference indicator in the Vienna consensus and defined as the number of mature (MII) oocytes at ICSI over the number of cumulus–oocyte complexes retrieved (ESHRE Special Interest Group of Embryology and Alpha Scientists in Reproductive Medicine, 2017). The indicator reflects the rate of mature oocytes from the oocytes collected and is a proxy indication of the effectiveness of ovarian stimulation (ESHRE Special Interest Group of Embryology and Alpha Scientists in Reproductive Medicine, 2017). Another definition was suggested to be the number of mature oocytes over the number of follicles ≥16 mm in stimulated cycles. This would make the indicator a relevant parameter to assess the competence of the clinician for deciding the time of trigger, with the cut-off of ≥16 mm based on a generally used criterion for trigger. It is clear that this indicator can only be calculated if the collected oocytes are fertilized by means of ICSI, which includes the removal of cumulus cells required to assess oocyte maturation.

*From the survey, there seemed to be a preference for the first definition of proportion of MII oocytes at ICSI, although both definitions were acceptable with over 70% agreement.*

#### OPU complication rate

Complications of OPU include bleeding (severe vaginal, intra-abdominal, or intra-peritoneal bleeding), infection (pelvic or ovarian abscess, pelvic infections), severe pain, or injury of pelvic structures. Complications related to sedation or anaesthesia have also been reported but are not considered a relevant PI for clinical practice in ART, as these complications occur with very low prevalence and are considered the responsibility of the anaesthetist. OHSS is excluded from the OPU complication rates, as it correlated to the ovarian stimulation and response, and it is suggested to be monitored as a separate PI.

It has been reported that the incidence and severity of complications during OPU are largely dependent on the training of the doctors (ESHRE Working Group on Ultrasound in ART *et al.*, 2019), patient characteristics, the number of retrieved oocytes, the duration of the OPU procedure, and the clinician's experience (Levi-Setti *et al.*, 2018). Concerning patient characteristics, complications are more prevalent in obese patients, or patients with co-morbidities (e.g. bleeding disorders, gynaecological pathologies) (Bennett *et al.*, 1993; Bodri *et al.*, 2008; Aragona *et al.*, 2011).

In the latest EIM data collection including 776 556 cycles, complications were reported in 1471 out of 918 159 (0.160%) cycles, including 983 bleedings, 117 infections, and 361 other complications (The European IVF-monitoring Consortium for the European Society of Human Reproduction and Embryology *et al.*, 2020). In a large observational study analyzing 23 827 OPUs from a single centre, the overall incidence of complications was 0.4% (Levi-Setti *et al.*, 2018), while a Cochrane meta-analysis reported an overall incidence of 0.72% of complications (Siristatidis *et al.*, 2018). Overall, the incidence of complications of OPU is small (ESHRE Working Group on Ultrasound in ART *et al.*, 2019).

Vaginal bleeding appears the most common complication of OPU, with a reported incidence ranging from 0.01% to 18.8%. This high difference may be attributed to a variation in the definition of vaginal bleeding. Peritoneal bleeding, representing a more serious complication of OPU, has a reported incidence of 0.05% to 0.35% (Liberty *et al.*, 2010; Aragona *et al.*, 2011; Levi-Setti *et al.*, 2018). Factors associated with an increased risk of bleeding include previous surgery (Levi-Setti *et al.*, 2018) and coagulation disorders (El-Shawarby *et al.*, 2004; Moayeri *et al.*, 2007). Pelvic organ (bowel, bladder, ureters) injury is a quite rare complication, with an incidence of 0.01–0.1% (Miller *et al.*, 2002; Fiori *et al.*, 2006; Ludwig *et al.*, 2006; Bozdag *et al.*, 2008; von Eye Corleta *et al.*, 2008), while pelvic infections range from 0.04% to 0.77% (Levi-Setti *et al.*, 2018; Ozaltin *et al.*, 2018). Severe pain requiring hospitalization is reported to occur in 0.06% to 0.7% of the cases (Ludwig *et al.*, 2006; Levi-Setti *et al.*, 2018; Ozaltin *et al.*, 2018). The majority of the reported serious complications during and after OPU in the literature are case reports.

For calculating the complication rate, a subgroup calculation in patients with normal BMI and no comorbidities can be suggested. All severe complications should be registered according to local regulations. A detailed medical history and a risk assessment are recommended prior to OPU.

Owing to the low prevalence of complications, and the absence of agreement on what entails a complication, this indicator has very little value in benchmarking. However, the PI is important for internal monitoring purposes.

*In the survey, the complication rate after OPU was considered a relevant PI. The complication rate after OPU should be calculated as the number of (any) complications that require an (additional) medical intervention or hospital admission (apart from OHSS) over the number of OPUs performed. Suggestions in the survey for also including mild bleeding and pain in the complication rate were balanced against comments on the feasibility of collecting the data on minor complications.*

### Indicators for ET and pregnancy

An ART treatment culminates in the ET procedure. The success of this procedure, and, thus, the success of the ART cycle as a whole, is affected by several factors, including ART regulations and ET policies, patient characteristics, ovarian stimulation, embryo quality (e.g. the number of top-quality embryos), and by ET operator skills and technique. In fact, it has been shown that notwithstanding patient characteristics and embryo quality, the main determinant of a successful ET is the operator (Coroleu *et al.*, 2002), and training for ET is necessary to reach competence (Lopez *et al.*, 2014). The success of the ET procedure can be assessed by means of IR (ESHRE Special Interest Group of Embryology and Alpha Scientists in Reproductive Medicine, 2017) and clinical pregnancy rate (%CPR), while ectopic and multiple pregnancy rates can be monitored to assess the safety of the procedure. Considering all the potential variables affecting ET outcome, the definition of global PIs for assessing operator skills in ET is a challenging task.

With regards to the ET policy, elective single ET (eSET) is considered the preferable route towards the key objective of ART, which is the birth of a healthy singleton child. For this reason, ESHRE is drafting an evidence-based guideline specifically on eSET. In the present document, the working group decided, based on the currently available evidence, to include multiple pregnancy rate as an indicator reflecting the transfer policy.

#### Clinical pregnancy rate (%CPR)

Clinical pregnancy is a commonly used criterion for measuring the effectiveness of ART, despite not being the final objective of the procedure. However, CPR is associated with clinician skills and, therefore, relevant to be used as the main PI for ET. There are many different outcomes and definitions used in clinical trials (CP, ongoing pregnancy, vital pregnancy) and this complicates the synthesis of evidence (Wilkinson *et al.*, 2016). Clinical pregnancy is defined as a pregnancy confirmed on US by visualization of one or more gestational sacs or definitive clinical signs of pregnancy (Zegers-Hochschild *et al.*, 2017). Without specification of the location of the pregnancy, this definition includes ectopic pregnancy (Zegers-Hochschild *et al.*, 2017; The ESHRE Guideline Group on Ectopic Pregnancy, 2020). For CPR, the International Committee for Monitoring Assisted Reproductive Technologies (ICMART) glossary suggests it can be calculated with different denominators (initiated, aspirated, or ET cycles). When reporting CPR values, the reference population and denominator should be specified (Zegers-Hochschild *et al.*, 2017). The relevance and challenges of each denominator are described in Table IV.

**Table IV:** Relevance and challenges of using different denominators in the definition of clinical pregnancy rate.

Denominator	Relevance	Challenges	Comments with regards to data collection	Comments with regards to PI calculations
**Per initiated cycle**	It assesses the probability of a successful ART procedure. The estimation is often made on the basis of the group of all patients starting treatment (intention-to-treat principle).	It cannot be used in cases of segmented cycles when all oocytes or embryos are cryopreserved for use and ET is performed in one of the future cycles.	Many registers do not record the start of the controlled ovarian stimulation (COS), and only report on cycles where COS ends with OPU.	Overlap with the PI—cycle cancellation rate (prior to OPU)
**Per OPU (i.e. per aspirated cycle)**	It assesses the probability of a successful ART procedure.	It cannot be used in cases of segmented cycles when all oocytes or embryos are cryopreserved for use and ET is performed in one of the future cycles.		
**Per embryo transfer**	It assesses the probability of a successful ET	The analysis lacks all cycles without ET, which consequently results in a seemingly higher effectiveness of ART.	It omits all (unsuccessful) cycles with no ET. The result is especially high when pregnancy, rather than live birth, is the numerator.	Important for calculation of individual PIs for clinicians.

PI, performance indicator.

CPR is a measure of efficacy, and the parameter does not consider aspects of safety, either ectopic or multiple pregnancies. Consequently, when used independently and without additional data, the indicator may incorrectly favour multiple ET strategies.

*The survey showed large agreement on considering CPR a relevant parameter to evaluate ET and on the definition, i.e. the number of pregnancies (diagnosed by ultrasonographic visualization of one or more gestational sacs or definitive clinical signs of pregnancy) over the number of ET cycles. Consistently, 69.19% of the respondents preferred per ET as the denominator for CPR in a specific question on the topic, while an additional 4.32% suggested calculating per ET and per OPU. Other suggested denominators included per OPU, per started cycle, per embryo transferred, and per patient. Per ET was considered the most appropriate denominator when using the indicator to assess operator skill. Clinical pregnancy is dependent on other factors, in addition to the operator skill, as discussed above, and using a reference population can be useful to perform meaningful comparisons.*

Using published data to deduce benchmark values for CPR or other indicators is problematic towing to lack of standardization in the definitions of the different parameters described. The lack of standardization is linked to the numerator (beta-hCG without ultrasonography validation, evidence of gestational sack, or evidence of foetal heartbeat) and the denominator (the number of started cycles, OPUs, or ETs). Furthermore, the recent shift in clinical management towards freeze-all cycles (up to 6.6% in the latest EIM report), either to prevent OHSS or as a strategy to improve clinical outcomes, resulted in more frozen and less fresh ETs (The European IVF-monitoring Consortium for the European Society of Human Reproduction and Embryology. *et al.*, 2020). Moreover, the recent increase in the number of natural or modified natural cycles performed, followed by embryo accumulation (mainly employed for patients with diminished ovarian reserve, or as part of regular strategy in some centres), makes treatment success calculations more difficult. A recent publication showed the impact of such changes in ART practice on CPR when comparing outcomes from fresh ET between 2004 and 2016 (Gleicher *et al.*, 2019). The impact of freeze-all strategies on overall clinical performance is to be clarified.

CPRs are included in international data collection, including the EIM data collection (The European IVF-monitoring Consortium for the European Society of Human Reproduction and Embryology *et al.*, 2020). Although mean values are available, the range is very wide owing to differences in patient characteristics (e.g. maternal age), national legislation (different techniques allowed), reimbursement, and clinical practice (e.g. number of embryos transferred and/or embryo developmental stage during transfer). As a result of this heterogeneity, data inconsistency, absence of data validation, and errors in data collection, it was deemed impossible to define competence and benchmark values for ET outcomes that could comprehensively apply to European clinics.

*The survey showed that around three-quarters of respondents agreed that benchmarks and competence levels for CPRs (and MPRs) should be defined at a local level (e.g. national), to minimize the effects of institutional or legislative differences on clinical outcomes.*

In addition to differences between countries, variation in reported CPR values can be attributed to the origin of the oocytes and the type of cycle (fresh or frozen). Therefore, it is recommended to assess CPR in the reference population (fresh ET with own oocytes), but also in frozen ET (FET) cycles with own oocytes, fresh ET after oocyte donation, and FET after oocyte donation. For those clinics with enough volume of treatments performed, it is recommended to split all four mentioned categories for cleavage and blastocyst ETs, as results can be significantly different between these groups.

#### Ectopic pregnancy

An ectopic pregnancy (i.e. a pregnancy outside the uterine cavity, diagnosed by US, surgical visualization or histopathology (Zegers-Hochschild *et al.*, 2017; The ESHRE Guideline Group on Ectopic Pregnancy, 2020)) is a pregnancy complication that has a slightly higher incidence in ART pregnancies compared with natural ones (Farquhar, 2005). This could be related to the existence of a previous pathology (e.g. pelvic inflammatory disease), infection at the time of the procedure, or embryo characteristics. Ectopic pregnancy appears to occur less frequently with frozen versus fresh ET (Xing *et al.*, 2018), and when a single frozen blastocyst is transferred compared to transfer of a cleavage-stage embryo(s) (Li *et al.*, 2015).

*The survey showed that less than half of the respondents considered ectopic pregnancy rate as a valuable PI for ET and one-third of them considered ectopic pregnancies not associated with ET technique or operator skills. The ectopic pregnancy rate was, therefore, not considered a relevant PI. The definition of ectopic pregnancy rate was accepted, and it should be calculated as the number of pregnancies outside the uterine cavity over the number of pregnancies.*

#### LBR, cumulative delivery rate and MPR as measures of performance in clinical practice in ART

A singleton live full-term healthy baby is the most relevant standard of success in ART (Min *et al.*, 2004). As such, LBR, cumulative delivery rate (cumDR), and MPR can be considered the ultimate KPIs in ART clinical practice. However, these parameters are highly dependent on local legislation with regards to restrictions on the number of embryos to be transferred, freeze-all strategies and reimbursement rules based on the number of initiated cycles versus the number of OPU.

Although highly relevant for trends evaluation, patient communication, and for monitoring the overall quality of the ART clinic, cumDRs and LBRs can only be calculated from retrospective data and cannot be used for monitoring clinical practice in ET.

LBR is defined as the number of deliveries that resulted in at least one live birth, divided by the number of cycles, expressed as a percentage. In the case of ART interventions, the latter cycles can be initiated cycles, cycles with oocyte aspiration, or cycles with ET. It is, thus, important that when delivery rates are reported, the denominator is specified (Zegers-Hochschild *et al.*, 2017). LBR is a generally accepted and important parameter for measuring ART success although it is a parameter that is related to many factors, even apart from the ART.

In the ICMART glossary, the cumDR is defined as the number of deliveries with at least one live birth resulting from one initiated or aspirated ART cycle, including all cycles in which fresh and/or frozen embryos are transferred, until one delivery with a live birth occurs or until all embryos are used, whichever occurs first. The delivery of a singleton, twin, or other multiples is registered as one delivery. In the absence of complete data, the cumDR is often estimated (Zegers-Hochschild *et al.*, 2017). Differently from ICMART, EIM defines the cumDR as the sum of all deliveries coming from fresh and frozen ET within 1 year from OPU, with the number of OPUs as the denominator (The European IVF-monitoring Consortium for the European Society of Human Reproduction and Embryology *et al.*, 2020). As the follow-up of individual patients over several years is difficult, EIM limits the time period for this parameter to 1 year and estimates the cumDR from deliveries and OPUs within the same year (EcumDR). The cumDR is an estimation from cross-sectional data and can be used, for instance, to assess the impact of the freeze-all technique on delivery rates. The cumDR after freeze-all cycles should preferably be considered separately from the cumDR after a fresh ET and all frozen transfers from the same OPU.

Since 2016, the EIM reports a cumDR (fresh and frozen) per aspiration. The cumDR was 29.6% for the countries that were able to provide data (The European IVF-monitoring Consortium for the European Society of Human Reproduction and Embryology *et al.*, 2020). One obvious limitation of this report is that not all FETs from freeze-all cycles could be completed within the 1-year time frame and were, therefore, not considered.

Multiple pregnancy or gestation is defined as a pregnancy with more than one embryo or foetus (Zegers-Hochschild *et al.*, 2017). Multiple pregnancy is the most frequent and most serious iatrogenic complication in ART procedures (Evers, 2002). The EIM data collection reporting 2016 shows large differences in MPRs across European countries, ranging from 1.1% to 35.7% (The European IVF-monitoring Consortium for the European Society of Human Reproduction and Embryology *et al.*, 2020). Lowering the occurrence of multiple pregnancies or deliveries has been advocated so as to increase ART safety and effectiveness (Min *et al.*, 2004), although not without unitary support (Braakhekke *et al.*, 2015).

*The survey respondents agreed on monitoring the occurrence of multiple pregnancies for performance evaluation. The vast majority of survey respondents agreed that MPR should be calculated as the number of multiple gestations over the number of pregnancies.*

### Training and competence

Ovarian stimulation, OPU, and ET must be performed by clinicians who are competently trained in reproductive medicine. In addition to training, competence in a certain procedure should be maintained.

#### Training

In some countries, fertility specialists or nurses can be trained to perform OPU and ET procedures, but there are currently no generally accepted minimal requirements for training. The Royal College of Obstetricians and Gynaecologists (RCOG) subspecialty curriculum does not contain any specific minimum number of procedures to be performed (RCOG, 2015), nor does the recent American Institute of Ultrasound in Medicine (AIUM) Practice Parameter for Ultrasound Examinations in Reproductive Medicine and Infertility (American Institute of Ultrasound in Medicine, 2017).

For safety reasons, and wherever feasible, a simulator could be the initial part of structured training for novices who want to perform these procedures, enabling them to acquire basic skills and to reach a predefined level of performance in a safe and controlled environment, before applying the procedure to patients (Soave *et al.*, 2019).

For OPU, the number of procedures to be performed while in training to reach the minimum criteria for competency was set to be a minimum of 30 OPUs under supervision, but this can vary depending on the type of training, background, and progress of trainees. In addition, at least 50 OPUs should be performed independently before the acquirement of the full qualification (ESHRE Working Group on Ultrasound in ART *et al.*, 2019). Proficiency has been defined as retrieving ≥80% of the oocytes compared with a senior operator performing OPU in the contralateral ovary (Dessolle, 2014).

Similar criteria were adopted by the European Fellows of Reproductive Medicine (EFRM) certification scheme. The number of procedures to be completed for training is listed in Table V. It is recommended to finalise training within a period of 2 years.

**Table V:** Number of procedures to be completed for training.

Procedure	Number of procedures to be completed for training (*within a period of 2 years)*
**Ovarian stimulation and trigger**	100 cycles^*^
**Oocyte collection/OPU**	75^*^
**Embryo transfer**	75^*^

* The numbers are those proposed by the working group, and should be applied in consideration that they were challenged in the survey.

#### Competence

After the training phase, maintaining competence or skills is essential. Criteria for assessing proficiency/competency on the technical aspects of OPU were recently included in a paper on good practice recommendations for OPU (ESHRE Working Group on Ultrasound in ART *et al.*, 2019) based on Dessolle, (2014) and Panayotidis (2017). Criteria for assessing proficiency/competency on the technical aspects of ET have not been described, but suggested criteria are pregnancy rates, number of ETs performed relative to the size of the clinic, and ectopic pregnancy rates.

In addition to competence for performing the procedure, a competence level can also be defined for teaching. The latter is not covered in the current paper.

Competence should be assessed regularly through peer to peer observational audits. The frequency of these audits of practice should be decided within the team. It is estimated that a procedure needs to be performed on average 200 times to achieve proficiency. However, rather than focussing on a number, PIs can be used to internally evaluate the maintenance of skills. PIs are essential to managing the performance of staff over time: it is all about managing the gap between expected performance (the PI) and actual performance (the result or measure). Once the gap between a benchmark and the actual performance is identified, it is vital to put a specific plan in place to reduce the gap. It makes no sense to establish a benchmark and measure performance against it if no action is taken to act upon the information gathered (Taylor, 2016).

The survey results showed overall an acceptance of using PIs to internally evaluate the maintenance of skills. The proposed values, as displayed in Table V, were challenged. Respondents stated that some of these targets might not be achievable within the differently structured training set up, suggesting more flexibility on the requirements. Fewer respondents suggested increasing the suggested number of procedures to be completed for training or competence. In addition, it was highlighted that the overall number of procedures appeared to be slightly overestimated, in particular for the ovarian stimulation.

## Conclusion

The current paper recommends six PIs to be used for monitoring clinical work in ART in ovarian stimulation, monitoring of ovarian stimulation, trigger and OPU, and ET and pregnancy. Furthermore, training and competence in relation to PIs are discussed. No PIs were defined for the first step in a standard ART process; diagnosis of infertility and indications for ART treatment. PIs for the laboratory procedures have been previously defined (ESHRE Special Interest Group of Embryology and Alpha Scientists in Reproductive Medicine, 2017).

In addition to the working group members and published data, the opinion of the stakeholders involved in the survey was vital in defining relevant and acceptable PIs for clinical work. Overall, the indicators and their definition as drafted by the working group were considered acceptable by stakeholders that participated in the online survey. The PIs defined in the current paper are either associated only with the performance of the clinician and clinic, or they are associated with outcomes that depend both on the clinic and the laboratory, and both share responsibility for results.

The PIs complement the earlier defined indicators for the ART laboratory (ESHRE Special Interest Group of Embryology and Alpha Scientists in Reproductive Medicine, 2017). Together, both sets of indicators aim to enhance the overall quality of the ART practice, and should be part of the total quality management of ART centres. They are important and can be used for education during subspecialty training. Future developments and shifts in ART clinical practice, such as eSET and the freeze-all technique, may warrant an update of the defined PIs and the suggested competence and benchmark values. The latter values are now based on the literature and consensus. However, ideally they should be defined on a local level; therefore, national and by extension international registries should be encouraged to standardise data collection, in line with defined ART clinic PIs and laboratory KPIs to enable the collected data to be used for the derivation of PI standard values. These initiatives will eventually lead to more functional competence and benchmark levels.

## Data availability

The data underlying this article are available in the article and in its online supplementary material.

## Supplementary Material

Supplementary_figure_S1

Supplementary_Table_SI
