# Supplementary figures and images for: The Maribor consensus: report of an expert meeting on the development of performance indicators for clinical practice in ART
Source: Hum Reprod Open. 2021 Jul 3;2021(3):hoab022. doi: 10.1093/hropen/hoab022 (PMC8254491; doi:10.1093/hropen/hoab022)

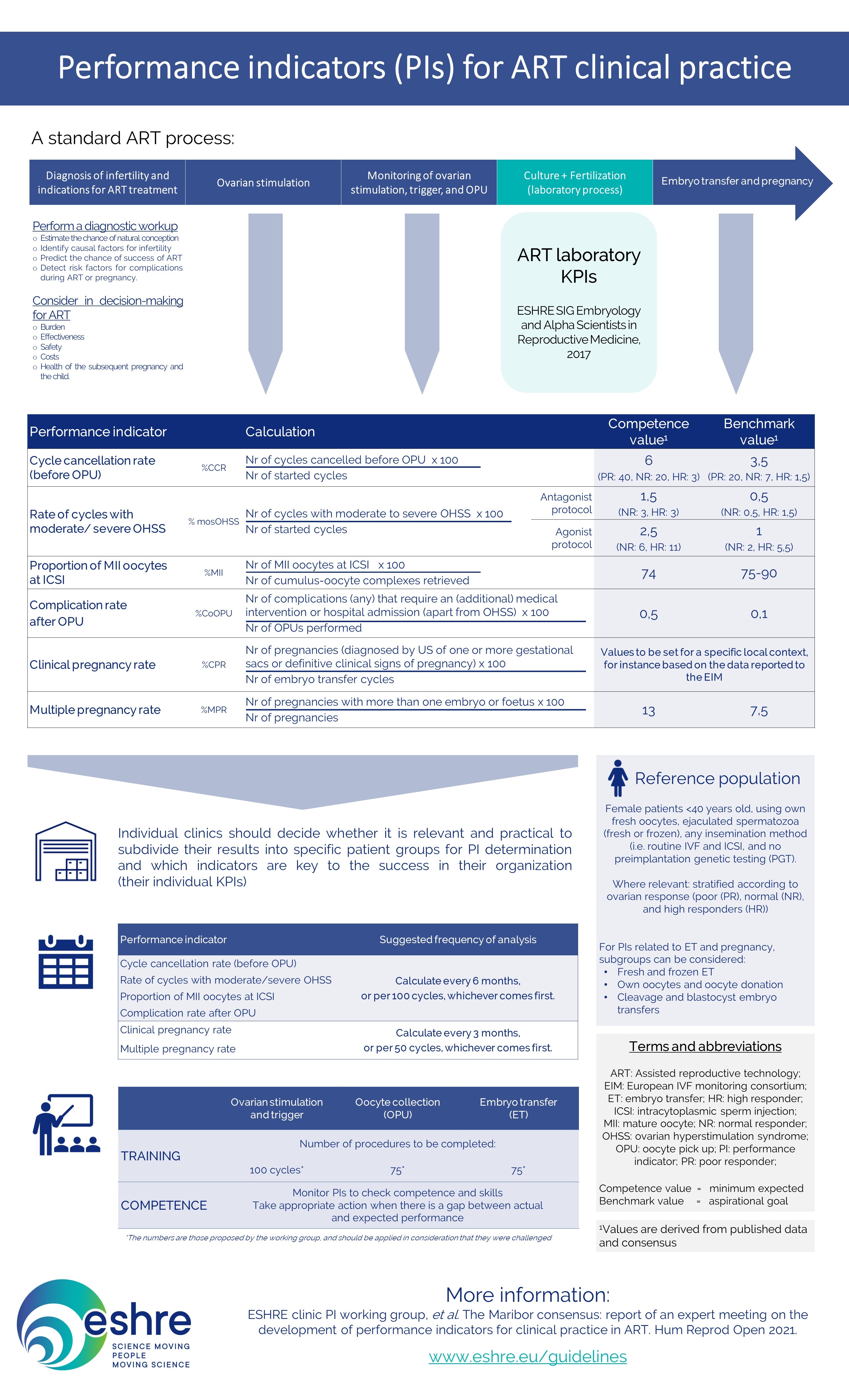

Supplement: Supplementary_figure_S1 [file supplementary_figure_s1.jpeg]
