# Supplementary material for: The Maribor consensus: report of an expert meeting on the development of performance indicators for clinical practice in ART
Source: Hum Reprod Open. 2021 Jul 3;2021(3):hoab022. doi: 10.1093/hropen/hoab022 (PMC8254491; doi:10.1093/hropen/hoab022)
Supplement: Supplementary_Table_SI [file supplementary_table_si.docx]

**Supplementary Table SI** Overview of the results of the survey with all statements and the percentages of agreement.

| **Statement** | **Nr of replies** | **Strongly agree** | **Agree** | **Neither agree nor disagree** | **Disagree** | **Strongly disagree** | **JUDGEMENT** |
| --- | --- | --- | --- | --- | --- | --- | --- |
| **Diagnosis and indications for ART treatment** | | | | | | | |
| Fertility work up should aim at establishing a prognosis of the residual chance of spontaneous conception, causal factors for infertility and the individual prognosis and risks for treatment and pregnancy. | 221 | 66.97 | 31.22 | 1.36 | 0.45 | 0.00 | ACCEPTED |
| Treatments other than by ART should be considered, when feasible, for patients with certain disorders amenable to other treatments. | 222 | 55.41 | 38.74 | 5.86 | 0.00 | 0.00 | ACCEPTED |
| When indicating ART, four treatment dimensions should be considered: burden, effectiveness, safety and costs (Dancet, 2014). | 221 | 60.63 | 33.03 | 5.43 | 0.90 | 0.00 | ACCEPTED |
| Expected benefits should be weighed against risks and burden of treatment, also taking the health of the subsequent pregnancy and the child into account. | 220 | 60.45 | 33.64 | 3.64 | 1.36 | 0.91 | ACCEPTED |
| ART should only be considered for cases with no alternative treatment of less invasiveness, burden, risks and costs. | 219 | 31.05 | 38.36 | 16.44 | 12.33 | 1.83 | DEBATABLE |
| **Ovarian stimulation** | | | | | | | |
| Cycle cancellation rate (before oocyte pick-up [OPU]) is a relevant parameter to measure performance in ovarian stimulation. | 206 | 26.21 | 45.63 | 17.48 | 8.25 | 2.43 | ACCEPTED |
| Cycle cancellation rate (before OPU) should be calculated as the number of cycles cancelled before OPU over the number of started cycles. | 205 | 44.88 | 44.88 | 7.32 | 1.46 | 1.46 | ACCEPTED |
| Cycle cancellation rate (before OPU) should be calculated separately for poor responders, normal responders and high responders. | 205 | 36.59 | 37.56 | 12.68 | 9.27 | 3.90 | ACCEPTED |
| Rate of cycles with moderate/severe ovarian hyperstimulation syndrome [OHSS] is a relevant parameter to measure performance in ovarian stimulation. | 206 | 36.89 | 45.63 | 10.68 | 4.85 | 1.94 | ACCEPTED |
| Rate of cycles with moderate/ severe OHSS should be calculated as the number of cycles with moderate to severe OHSS (as defined by the *European IVF-monitoring Consortium: EIM*) over the number of started cycles. | 204 | 37.75 | 50.98 | 8.82 | 1.96 | 0.49 | ACCEPTED |
| Rate of cycles with moderate/severe OHSS should be calculated separately for agonist and antagonist cycles. | 205 | 31.71 | 40.98 | 14.63 | 7.80 | 4.88 | ACCEPTED |
| Rate of cycles with moderate/severe OHSS should be calculated separately for poor responders, normal responders and high responders. | 206 | 22.33 | 34.47 | 21.84 | 15.53 | 5.83 | DEBATABLE |
| **Monitoring of ovarian stimulation, trigger and oocyte pick-up** | | | | | | | |
| Oocyte retrieval rate is a relevant parameter to measure performance in monitoring of ovarian stimulation, trigger and OPU. | 192 | 37.50 | 45.83 | 9.38 | 3.65 | 3.65 | ACCEPTED |
| Oocyte retrieval rate should be calculated as the number of oocytes retrieved over the number of follicles (>10mm) on the day of trigger. | 193 | 22.28 | 40.93 | 12.44 | 20.21 | 4.15 | DEBATABLE |
| Number of mature (MII) oocytes per number of follicles (≥ 16mm) is a relevant parameter to measure performance in monitoring of ovarian stimulation, trigger and OPU. | 193 | 37.31 | 43.52 | 9.84 | 6.22 | 3.11 | ACCEPTED |
| Number of mature (MII) oocytes per number of follicles (≥ 16mm) should be calculated as the number of MII oocytes at ICSI over the number of follicles (≥16mm) on the day of trigger. | 192 | 23.44 | 50.00 | 13.54 | 8.33 | 4.69 | ACCEPTED |
| The oocyte maturation rate should be defined as the number of MII oocytes at ICSI over the number of cumulus-oocyte complexes retrieved (as in the Vienna consensus) | 193 | 44.04 | 40.93 | 10.36 | 3.63 | 1.04 | ACCEPTED |
| Complication rate after OPU is a relevant parameter to measure performance in monitoring of ovarian stimulation, trigger and OPU. | 193 | 32.12 | 39.38 | 16.58 | 10.36 | 1.55 | ACCEPTED |
| Complication rate after OPU should be calculated as the number of complications (any) that require an (additional) medical intervention or hospital admission (apart from OHSS) over the number of OPUs performed. | 193 | 42.49 | 43.52 | 11.40 | 2.07 | 0.52 | ACCEPTED |
| **ET and Pregnancy** | | | | | | | |
| Clinical pregnancy rate is a relevant parameter to measure performance in embryo transfer (ET) and pregnancy. | 186 | 51.08 | 36.02 | 5.38 | 6.45 | 1.08 | ACCEPTED |
| Clinical pregnancy rate should be calculated as the number of pregnancies (diagnosed by ultrasonographic visualization of one or more gestational sacs or definitive clinical signs of pregnancy) over the number of ET cycles. | 186 | 47.31 | 38.71 | 5.38 | 6.45 | 2.15 | ACCEPTED |
| Multiple pregnancy rate is a relevant parameter to measure performance in ET and pregnancy. | 186 | 46.77 | 27.42 | 12.90 | 9.68 | 3.23 | ACCEPTED |
| Multiple pregnancy rate should be calculated as the number of pregnancies with more than one embryo or fetus over the number of pregnancies. | 186 | 52.15 | 40.86 | 3.23 | 2.15 | 1.61 | ACCEPTED |
| Ectopic pregnancy rate is a relevant parameter to measure performance in ET and pregnancy. | 186 | 18.82 | 26.34 | 21.51 | 25.81 | 7.53 | DEBATABLE |
| Ectopic pregnancy rate should be calculated as the number of pregnancies outside the uterine cavity over the number of pregnancies. | 185 | 44.32 | 47.03 | 4.86 | 2.70 | 1.08 | ACCEPTED |
| Benchmark and competence values for clinical pregnancy rate should be set for a specific local context, for instance from the data reported to the EIM for the same country | 186 | 27.42 | 50.00 | 13.98 | 5.38 | 3.23 | ACCEPTED |
| Benchmark and competence values for multiple pregnancy rate should be set for a specific local context, for instance from the data reported to the EIM for the same country | 185 | 28.65 | 45.95 | 13.51 | 8.65 | 3.24 | ACCEPTED |
| Should clinical pregnancy rate be measured per ET or per OPU? | 185 | 69.19% preferred *per ET* with an additional 4.32% suggested calculating *per ET* and *per OPU* . Other suggested denominators included *per OPU* (10.27%), *per started cycle* (8.65 %), *per embryo transferred* (2.70%), *per patient*  (1.08%) and other replies (3.78%). | | | | | na |
| **Training and competence** | | | | | | | |
| Number of procedures to be completed for training and competence should be: for induction of ovulation and trigger, 100 cycles for training); for IUI, 100 procedures for training; for oocyte collection, 75 procedures for training, 200 for competency; and for ET, 75 procedures for training, 200 for competency. | 181 | 19.34 | 41.44 | 19.34 | 17.68 | 2.21 | DEBATABLE |
| Performance indicators can be used to internally evaluate maintenance of skills. | 181 | 46.41 | 46.96 | 4.42 | 1.66 | 0.55 | ACCEPTED |
| **General assessment** | | | | | | | |
| The list of indicators for clinical practice in ART is acceptable; cycle cancellation rate (before OPU), rate of cycles with moderate/severe OHSS, oocyte retrieval rate, number of mature (MII) oocytes per number of follicles (≥ 16mm), complication rate after OPU, clinical pregnancy rate, multiple pregnancy rate, and ectopic pregnancy rate . | 169 | 20.71 | 58.58 | 11.83 | 7.10 | 1.78 | ACCEPTED |
| Can you think of any additional performance indicators that should be added? | 165 | 77.6% of responders replied that they could not think of additional *performance indicators* to be added. Suggestions for additions (suggested more than once) included OPU complication rate, number of embryos, implantation rate, pregnancy loss rate, biochemical pregnancy rate, (cumulative) pregnancy rate , (cumulative) live birth rate | | | | |  |
| Indicators for clinical practice in ART should be calculated every 6 months or per 100 cycles, whatever comes first, except for clinical and multiple pregnancy rates , which should be calculated every 3 months, or per 50 cycles, whatever comes first. | 168 | 23.81 | 59.52 | 8.33 | 5.95 | 2.38 | ACCEPTED |
